# Cell Fate Decisions Within the Vascular Cambium–Initiating Wood and Bast Formation

**DOI:** 10.3389/fpls.2022.864422

**Published:** 2022-04-25

**Authors:** Aylin S. Haas, Dongbo Shi, Thomas Greb

**Affiliations:** ^1^Centre for Organismal Studies (COS), Heidelberg University, Heidelberg, Germany; ^2^RIKEN Center for Sustainable Resource Science (CSRS), Tsurumi-Yokohama, Japan; ^3^Japan Science and Technology Agency (JST), Precursory Research for Embryonic Science and Technology (PRESTO), Kawaguchi, Japan

**Keywords:** cambium, cell fate specification, gene regulatory network (GRN), wood formation, stem cell

## Abstract

Precise coordination of cell fate decisions is a hallmark of multicellular organisms. Especially in tissues with non-stereotypic anatomies, dynamic communication between developing cells is vital for ensuring functional tissue organization. Radial plant growth is driven by a plant stem cell niche known as vascular cambium, usually strictly producing secondary xylem (wood) inward and secondary phloem (bast) outward, two important structures serving as much-needed CO_2_ depositories and building materials. Because of its bidirectional nature and its developmental plasticity, the vascular cambium serves as an instructive paradigm for investigating principles of tissue patterning. Although genes and hormones involved in xylem and phloem formation have been identified, we have a yet incomplete picture of the initial steps of cell fate transitions of stem cell daughters into xylem and phloem progenitors. In this mini-review perspective, we describe two possible scenarios of cell fate decisions based on the current knowledge about gene regulatory networks and how cellular environments are established. In addition, we point out further possible research directions.

## Introduction

**FIGURE 1:**
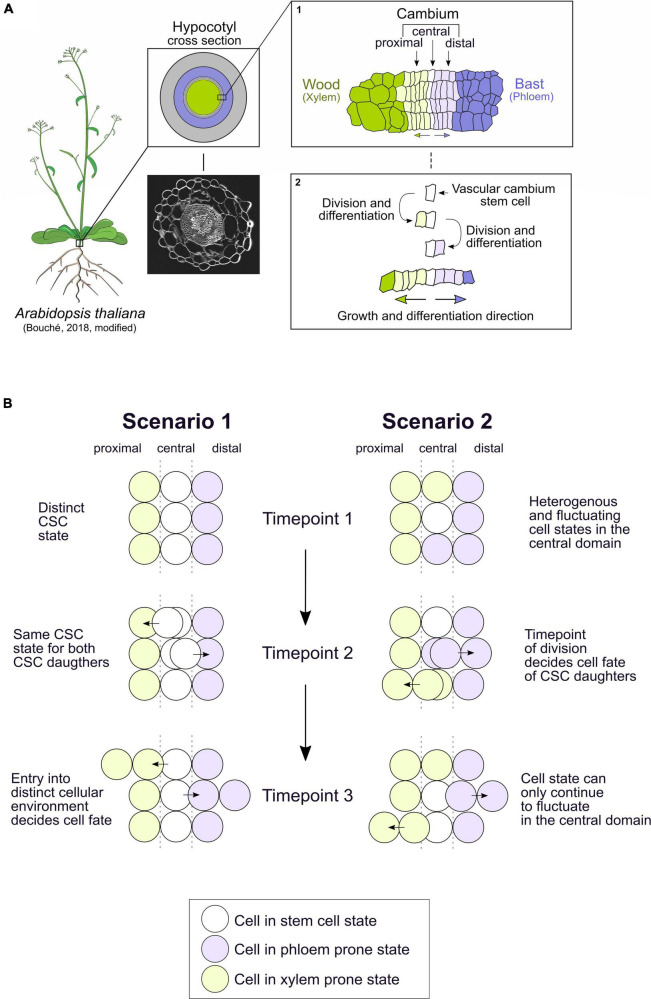
The vascular cambium in the hypocotyl of *Arabidopsis thaliana* produces xylem and phloem in a bidirectional manner. The cell fate decision of the bifacial vascular cambium stem cell could be triggered by different mechanisms. **(A)** The hypocotyl of *Arabidopsis thaliana* is a hotspot of vascular cambium activity, producing xylem tissue inwards, here in dark green, and phloem tissue outwards, here in dark purple. A confocal image of a hypocotyl section with stained cell walls (Direct Red 23) is shown. Inset 1: The cambium itself can be divided into the central domain, harboring the cambium stem cells (CSCs) (white), the proximal domain (light green), and the distal domain (light purple). Inset 2: So far, it is assumed that the vascular cambium stem cell divides and then differentiates into a xylem progenitor in the proximal domain or a phloem progenitor in the distal domain. After this initial division, the progenitor cells only divide once or twice before they fully differentiate into xylem or phloem cell types with increasing distance to the central cambium domain. **(B)** The described process in Figure 1A inset 2 could be explained by two scenarios of cell fate decisions which might take place in the vascular cambium. In the first scenario, the CSCs have a distinct stem cell state in the central cambium domain which is robust over time (timepoint 1). Upon division, both CSC daughter cells have the same CSC state (timepoint 2). As one CSC daughter leaves the central domain into the proximal or distal domain, it is exposed to a different cellular environment which dictates its direction of differentiation into xylem or phloem (timepoint 3). In the second scenario, the state of cells in the central domain is heterogeneous and fluctuates between a CSC state and a xylem- or phloem-prone state (timepoint 1). Here, the timepoint of the division dictates whether xylem or phloem progenitor cells are produced in the central domain (timepoint 2). Only the daughter cell which remains in the central domain is still able to fluctuate between states, the cell that exits the central domain loses its capacity to fluctuate between cell states (timepoint 3). Arabidopsis drawing template by Bouché (2018).

Usually, multicellular organisms harbor pools of stem cells that maintain a pluripotent cell state at critical positions within their bodies. These stem cells produce new tissues consisting of differentiated cells performing specialized tasks. A central question in developmental biology is how stem cells know when and at which position they need to differentiate into a certain cell type. In plants, in particular, precise spatiotemporal regulation of differentiation is crucial, as the rigid cell wall prevents cellular migration. Due to its strictly bidirectional mode of tissue production, the vascular cambium (hereinafter called cambium), has a far simpler setup than other plant stem cell niches harboring multidirectional modes (Greb and Lohmann, 2016). Hence, the cambium is ideal to tackle principles of cell fate decisions in plants and beyond. In many species, the cambium consists of a cylindrical domain of stem cells, producing wood inward and bast outward (Larson, 1994; Evert, 2006; Shi et al., 2019; Smetana et al., 2019). Wood (xylem) transports water and nutrients from roots to shoots and stabilizes the plant body based on exceptionally stiff cell walls produced by xylem cells, whereas bast (phloem) mainly transports sugars and signaling molecules from source to sink tissues. In the model plant *Arabidopsis thaliana*, the hypocotyl, the organ connecting the root and the shoot systems, is a hotspot of cambium activity (Figure 1A; Chaffey et al., 2002). It is to note that the cambium structure of *Arabidopsis* and tree species such as poplar are not identical. However, the development and regulation of radial growth is similar, hence, *Arabidopsis* can serve as a paradigm for radial plant growth (Barra-Jiménez and Ragni, 2017). Functionally, the cambium is divided into three domains: the proximal domain, in which xylem differentiation is initiated, the central domain that harbors a single layer of cambium stem cells (CSCs), and the distal domain where phloem initiation takes place (Figure 1A inset 1; Shi et al., 2019). During radial growth, the proliferative activity of CSCs determines the rate of xylem and phloem production, since xylem and phloem progenitors divide only once or twice before they fully differentiate (Figure 1A inset 2; Bossinger and Spokevicius, 2018; Shi et al., 2019; Smetana et al., 2019).

In this mini-review perspective, we present two possible scenarios of how cell fate decisions of the CSC daughters take place and discuss them in the light of recent findings on gene regulatory mechanisms and signaling regimes. We also examine the knowledge gap that needs to be filled in order to obtain a conclusive picture of how xylem and phloem formation is initiated. We mainly focus on the *Arabidopsis* hypocotyl, but also include findings on primary xylem and phloem formation in stems and roots. It is to note, however, that insights obtained from primary vascular development may not be transferable to the hypocotyl, as radial growth regulation between the various organs is partially different (Cammarata et al., 2019). For a more thorough overview of radial growth, the interested reader may consult extensive reviews published elsewhere (Fischer et al., 2019; Fukuda and Ohashi-Ito, 2019; Turley and Etchells, 2021).

## Two Scenarios for Cell Fate Decisions of Cambium Stem Cell Derivatives

It was postulated already 150 years ago that individual CSCs produce both the xylem and phloem and, therefore, have a bifacial character (Sanio, 1873; Larson, 1994). Only rather recently, however, detailed lineage tracing showed that indeed a single bifacial CSC produces xylem and phloem lineages (Bossinger and Spokevicius, 2018; Shi et al., 2019; Smetana et al., 2019). Still, it is largely unclear what determines the decision of CSC derivatives to differentiate into either xylem or phloem progenitor cells. Here, we present two possible scenarios differing in the spatiotemporal pattern of decision events (Figure 1B). It is important to mention that both scenarios are brought up for conceptual reasons and may not be mutually exclusive. In the first scenario, CSCs have a distinct and robust stem cell state in the central cambium domain, i.e., a stable transcriptome state and other stem cell-associated molecular attributes. Initially, each CSC daughter is in the same state as the CSC but, as it leaves the central domain, it is exposed to a different cellular environment with a distinct signaling regime and therefore enters xylem or phloem development. In the second scenario, CSCs are heterogeneous and fluctuate between the CSC state and a xylem- or phloem-prone state. In this scenario, depending on the state of the cells in the central domain at the time of division, xylem or phloem progenitor cells are produced. After division, when cells exit the central domain, they lose their ability to fluctuate to other states, whereas the cell which remains in the central domain can still fluctuate between all the cell states (Figure 1B). State fluctuation represents a way of creating cellular heterogeneity within animal embryonic stem cells (Okamoto et al., 2018; Huang et al., 2020) and noise in gene activity is often important for promoting cell fate transitions (Eldar and Elowitz, 2010), making the second scenario worth considering in the CSC context.

We believe that conceptualizing both the scenarios is fruitful as this process brings up several questions central for cambium regulation and stem cell biology in general: Which gene regulatory networks mediate the transition from a stem cell state to a xylem- or phloem-prone progenitor state and what are their spatiotemporal patterns? Moreover, which signals establish different cellular environments in the proximal, central, and distal cambium domain?

## Gene Regulatory Networks in Cambium Stem Cells

In both the aforementioned scenarios, the cells in the central domain of the cambium are pluripotent stem cells. In animals, gene regulatory networks (GRNs) mediating pluripotency in embryonic stem cells are centered around a handful of transcription factors (TFs) and are well understood (Nichols et al., 1998; Li and Izpisua Belmonte, 2018; Huang et al., 2020). In CSCs, however, it is still unclear which genes make up the “stemness” GRN. Plant stem cell specification and maintenance is generally carried out by conserved WUSCHEL-related homeobox (WOX) TFs, which already mark cell fate decisions during early embryonic states (Haecker et al., 2004; Dolzblasz et al., 2016). In the context of the cambium, WOX4 together with WOX14 promotes stem cell proliferation downstream of a CLAVATA3/EMBRYO SURROUNDING REGION-related (CLE) peptide-signaling cascade (Hirakawa et al., 2010; Suer et al., 2011; Etchells et al., 2013). This well-studied signaling cascade consists of the PHLOEM INTERCALATED WITH XYLEM (PXY) receptor and its ligands CLE41/CLE44, also named TRACHEARY ELEMENT DIFFERENTIATION INHIBITORY FACTOR (TDIF), and CLE42. CLE41/42/44 are expressed in the phloem and are thought to diffuse toward the CSCs, bind to the PXY receptor and, ultimately, elevate *WOX4* and *WOX14* gene activities (Ito and Fukuda, 2006; Fisher and Turner, 2007; Hirakawa et al., 2008; Etchells and Turner, 2010; Morita et al., 2016; Zhang et al., 2016).

**FIGURE 2:**
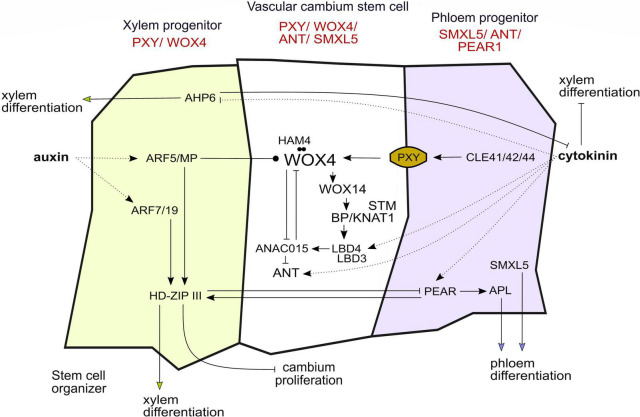
Gene regulatory networks and hormone signaling in the vascular cambium influence cell fate decisions and stem cell dynamics. The gene regulatory network in the vascular cambium acts cell autonomously and non-cell autonomously, favoring cambium stem cell proliferation or differentiation of the progenitor cells into xylem and phloem. So far, the picture of the “stemness” gene regulatory network is still not complete. The genes depicted in red are the main markers of expression used to differentiate between the cambium domains. Arrows indicate activation or inhibition, respectively. Dotted lines indicate the action of hormones, the arrow ending with a dot indicates attenuation of WOX4 activity through ARF5/MP. The dots between HAM4 and WOX4 indicate protein–protein interaction.

However, *WOX4* and *WOX14* expression alone is not the only factor maintaining CSCs since cambium activity is not completely abolished in *wox4* or *wox4*; *wox14* mutants (Hirakawa et al., 2010; Etchells et al., 2013). Looking at transcriptional markers, CSCs are marked by overlapping promoter activities of the *WOX4* and *PXY* genes, but also of *AINTEGUMENTA* (*ANT*) and *SUPPRESSOR OF MAX2 1-LIKE 5* (*SMXL5*) (Figure 2; Etchells et al., 2015; Shi et al., 2019, 2021; Smetana et al., 2019). *SMXL5* is involved in the phloem formation and is active in the distal cambium domain (Wallner et al., 2017, 2020; Shi et al., 2019) and the cytokinin-responsive TF ANT is required for normal radial growth in the root (Randall et al., 2015). As an important step toward the understanding of CSC regulation, a transcriptional regulatory network consisting of 13 genes including *WOX4*, *WOX14*, *ANT* as well as *BREVIPEDICELLUS/KNOTTED-1LIKE 1* (*BP/KNAT1*) has been constructed recently (Zhang et al., 2019). Using fluorescence-activated cell sorting of cells expressing a tagged cytokinin responsive TF at three stages of cambium development and subsequent genome-wide transcript profiling, candidate TFs for CSC regulation were identified and further validated (Zhang et al., 2019). Within the resulting network, *WOX4* suppresses the *NAC DOMAIN CONTAINING PROTEIN 15* (*ANAC015*) gene which, in turn, suppresses *ANT* (Figure 2). In addition, *WOX4* activates *BP/KNAT1* through *WOX14* and, based on mutant phenotyping, *WOX4* and *BP/KNAT1* were identified as major determinants of CSC activity (Zhang et al., 2019). Moreover, *BP/KNAT1* controls early events in vascular differentiation together with the *SHOOT MERISTEMLESS* (*STM*) gene (Liebsch et al., 2014). Another part of the CSC network are LOB DOMAIN-CONTAINING PROTEIN (LBD) TFs, of which LBD3 and LBD4 were shown to be important for CSC maintenance, proliferation, and vascular cell differentiation (Figure 2; Zhang et al., 2019; Smit et al., 2020; Ye et al., 2021). Furthermore, *LBD4* acts downstream of *WOX4, WOX14* and *BP/KNAT1* and regulates vascular cell number and organization (Zhang et al., 2019). Both *LBD3* and *LBD4* genes are cytokinin responsive and important factors in the regulation of cambium activity (Zhang et al., 2019; Smit et al., 2020; Ye et al., 2021). Furthermore, *HAIRY MERISTEM* (HAM) TFs positively influence the amount of cambium-derived tissues and, in particular, HAM4 and WOX4 proteins form complexes presumably in CSCs (Zhou et al., 2015).

In summary, *WOX4* and *WOX14* as well as *BP/KNAT1*, *STM*, *ANT*, and *LBDs* have emerged as master regulators of CSC activity. However, it becomes evident, that the results described earlier draw a yet incomplete picture of the “stemness” and “differentiation” GRNs regulating proliferation and cell fate decisions. Are identified genes continuously expressed and how does the network alter during xylem and phloem specification? With regard to the first scenario, assuming a robust stem cell state, the aforementioned regulators would be stably expressed in CSCs and all the genes involved in promoting differentiation would have to be stably counteracted in the central domain. In the second scenario, the transcriptome of the CSCs would fluctuate and thus would the genes which are mentioned earlier. Since some of the aforementioned genes promote xylem or phloem differentiation, it is possible that upon their activation CSCs switch to a xylem- or phloem-prone state (Figure 2).

This leads to a central question: Do CSCs have a unique transcriptome, and if so, how does their transcriptome differ from those of xylem and phloem progenitor cells? The course of differentiation from a vascular stem cell to differentiated protophloem sieve elements has only been described recently in the context of primary vascular development in the root (Roszak et al., 2021). Using lineage tracing, single-cell RNA sequencing (scRNA-seq) and pseudo time analysis, the authors show that there is a sharp and abrupt change in the cells’ transcriptome as they approach a differentiated state. Four so-called transcriptomic domains were identified, namely, “amplification,” “transition,” where lineage bifurcation takes place, “early differentiation” and “late differentiation.” The domains convert into each other through rapid developmental transitions based on GRNs with mutual inhibition modules. The stem cell produces transit amplifying cells which develop into cells that experience lineage bifurcation into procambium and metaphloem sieve elements. Lineage bifurcation is dependent on the activity of lineage-specific PHLOEM EARLY DNA-BINDING-WITH ONE-FINGER (PEAR) TFs which activate distinct *RHO OF PLANTS GUANINE NUCLEOTIDE EXCHANGE FACTOR* (*ROPGEF-ROP*) genes. *PEARs* promote phloem differentiation also through activation of the *ALTERED PHLOEM DEVELOPMENT* (*APL*) gene, encoding a member of the MYB TF family (Figure 2). In the stem cells and the transit amplifying cells, *PEAR* activity is suppressed through a reverse protein concentration gradient of the PLETHORA (PLT) TFs (Roszak et al., 2021). To summarize, these findings indicate that in the root, vascular stem cells, and phloem progenitors carry distinct transcriptomes and that the transition of transcriptomes is abrupt, favoring, in general terms, scenario 1.

Nevertheless, it is still unclear whether the transitions of the transcriptome, i.e., the fluctuation of cell states, can also directly take place in the central domain, which is described in the second scenario. But the strong upregulation of TFs such as WOX4 in the central domain suggests a distinct and robust cell state in the central cambium domain or at least a strong dominance of the CSC state. However, so far, there is no temporal CSC analysis with cellular resolution possible and no scRNA-seq datasets characterizing hypocotyl CSCs are available. To circumvent obstacles for life-cell imaging, fluorescent timers to track CSC dynamics over time could be used (Masuyama et al., 2019). Interestingly, mathematical modeling showed that gene activity noise among seemingly identical cells cannot be determined by only using gene expression data from scRNA-seq experiments, since changes in a particular cell over time are missing (Ham et al., 2021). Here, DNA barcoding in combination with scRNA-seq to trace individual cells may solve this issue (Zhang et al., 2020). Moreover, by including information about other molecules that are affected by the expression of a particular gene, it is possible to determine whether its activity variation is the result of intrinsic or extrinsic noises. Hence, creating a “pathway reporter” pair, such as mRNA and protein or nascent RNA and mRNA, may help to understand the source of CSC heterogeneity (Peterson et al., 2017; Ham et al., 2021).

## Establishment of Distinct Cellular Environments Through Plant Hormones

In the scenarios we introduced, the cellular environment dictates either the route of differentiation in the proximal or distal domain (scenario 1) or the cell states in the central domain (scenario 2). Cellular environments can be shaped by protein gradients as has been shown for CLE and PLT proteins (Fisher and Turner, 2007; Roszak et al., 2021). In addition, it is well established that plant hormones are essential for maintaining stem cell niches and for regulating stem cell differentiation (Greb and Lohmann, 2016). Most of the aforementioned genes are linked to plant hormones because they are either directly regulated by them or are downstream targets of other hormone-regulated genes. Here, we focus on the two hormones cytokinin and auxin since they have emerged as major regulators of stem cell niches (Bishopp et al., 2011a,b; Su et al., 2011; Salvi et al., 2020).

A widely discussed concept is that auxin gradients convey positional information along the cambium (Sachs, 1991; Uggla et al., 1996; Bhalerao and Fischer, 2014). Direct measurements in trees suggest that auxin levels are high in CSCs and drop toward more differentiated cells (Immanen et al., 2016). In turn, in *Arabidopsis*, auxin signaling seems relatively low in CSCs, though being required for their activity, and increases in differentiating xylem and phloem cells (Brackmann et al., 2018). The AUXIN RESPONSE FACTOR 5/MONOPTEROS (ARF5/MP) restricts the number of undifferentiated cambium cells and promotes the differentiation of CSCs into xylem cells. Furthermore, ARF5/MP directly targets *WOX4* as well as xylem- and phloem-related genes, suggesting a general role for ARF5/MP in promoting vascular differentiation (Brackmann et al., 2018). In the root, proximity to xylem progenitor cells, showing high levels of auxin signaling, is important to determine CSC activity, defining xylem progenitor cells as a CSC organizer (Smetana et al., 2019). It is thought that after CSC organizing cells have fully differentiated into xylem, newly produced CSC daughters take over the organizer role. Downstream of ARF5/MP, as well as of ARF7 and ARF19, HOMEODOMAIN LEUCIN ZIPPER III (HD-ZIP III) TFs determine the organizer role by promoting xylem differentiation (Figure 2; Smetana et al., 2019).

The concept of a stem-cell organizer implies that both the proximal organizer (xylem progenitor cells) and the CSCs hold distinct states and that these states are determined by hormone-signaling levels associated with relative cellular position. However, before the stem cell produces two daughter cells, it might encompass or fluctuate between both identities, the CSC and the xylem progenitor state. This fluctuation does not need to be based on different transcriptome states. Fluctuation could also be on the level of cell biological attributes such as endomembrane organization or the distribution of plasma membrane-associated proteins. In this context, it is important to note that we still do not know if CSCs are polarized along radial organ axes and if such a polarity influences cell fate decisions. Roszak et al. shed light on this issue in the root by showing that lineage bifurcation in phloem development is dependent on cell polarity acquired through ROPGEF-ROPs which help position the division plane (Roszak et al., 2021). In summary, auxin is a very important factor shaping the cellular environment of the cambium and promoting CSC activity and xylem cell differentiation.

In addition to auxin, cytokinin has been long demonstrated to be important for cambium regulation (Aloni, 1987). Local cytokinin biosynthesis is crucial for vascular development (De Rybel et al., 2014) and the hormone peaks in developing phloem cells (Immanen et al., 2016). Cytokinin also influences auxin transport and distribution by regulating the expression of *PIN-FORMED (PIN)* genes (Růzǐčka et al., 2009; Šimášková et al., 2015). In roots, a simple regulatory circuit, including cytokinin and auxin, regulates cell fate decisions and radial patterning during vascular development. In this case, cytokinin inhibits differentiation of the procambium into protoxylem. The protein HISTIDINE PHOSPHOTRANSFER PROTEIN 6 (AHP6) counteracts the influence of cytokinin in developing xylem cells and thereby promotes protoxylem formation, while being itself inhibited by cytokinin (Figure 2; Mähönen et al., 2006). Cytokinin also initiates the expression of *PEAR1* and *PEAR2* whose activity peaks in the phloem, more specifically, in protophloem sieve elements. HD-ZIP IIIs antagonize the effect of PEAR proteins and, together, both the regulatory groups define the boundary between dividing and non-dividing vascular cells in the root (Figure 2; Miyashima et al., 2019). Also DNA-BINDING-WITH ONE-FINGER 2.1 (DOF2.1), which is expressed in the outer procambium cells, is induced by cytokinin and specifically promotes proliferation in the outer procambium cells (Smet et al., 2019). Recently, Yang et al. proposed a model for the spatiotemporal control of cytokinin levels in the root: Auxin first increases cytokinin levels through activating a TF complex that induces genes which positively influence cytokinin levels in the xylem. The same TF complex also activates downstream processes that attenuate cytokinin levels in the procambium. This balancing ensures normal differentiation and proliferation of vascular cells (Yang et al., 2021). Taken together, cytokinin acts in the cambium to safeguard cell proliferation, to inhibit xylem differentiation and to promote phloem formation.

Auxin and cytokinin are two hormones which form highly interactive gradients of concentration and signaling, creating distinct cellular environments, initiating different downstream events in the respective cambium domains. With regard to the two proposed scenarios, auxin and cytokinin would either dictate proximal, central, or distal cell fates or limit the capacity of a cell to fluctuate between the cell states. In both scenarios, distinct auxin–cytokinin signaling levels may be important to ensure that proliferation only occurs in the central cambium domain. Knowing that transcriptome changes during lineage bifurcation are fairly rapid (Roszak et al., 2021), one could argue that these gradients must be sharp or that additional mechanisms are required for a fast and precise transition between cell states.

## Concluding Remarks

The vascular cambium consists of the bifacial stem cells in the central domain, producing xylem and phloem progenitors in a bidirectional manner. Gene regulatory networks must specifically convey either stemness or a xylem- or phloem-prone state, and these networks may be altered strictly in a spatial or also in a temporal mode. In this context, great progress has been made in identifying members of the gene regulatory network acting in CSCs providing a molecular signature whose dynamics is open for investigation. It can be expected that techniques such as scRNA-seq, temporal-resolved lineage tracing, and cell biological characterization of CSCs will substantially contribute to establish a more comprehensive understanding of an intriguing and highly relevant stem cell system.

## Author Contributions

ASH drafted the manuscript and prepared the figures. DS and TG reviewed and edited the manuscript. All authors contributed to the article and approved the submitted version.

## Conflict of Interest

The authors declare that the research was conducted in the absence of any commercial or financial relationships that could be construed as a potential conflict of interest.

## Publisher’s Note

All claims expressed in this article are solely those of the authors and do not necessarily represent those of their affiliated organizations, or those of the publisher, the editors and the reviewers. Any product that may be evaluated in this article, or claim that may be made by its manufacturer, is not guaranteed or endorsed by the publisher.

## References

[B1] Aloni R. (1987). Differentiation of vascular tissues. *Ann. Rev. Plant Physiol.* 38 179–204. 10.1146/annurev.pp.38.060187.001143

[B2] Barra-Jiménez A. Ragni L. (2017). Secondary development in the stem: when *Arabidopsis* and trees are closer than it seems. *Curr. Opin. Plant Biol.* 35 145–151. 10.1016/j.pbi.2016.12.002 28013083

[B3] Bhalerao R. P. Fischer U. (2014). Auxin gradients across wood – instructive or incidental? *Physiol. Plant.* 151 43–51. 10.1111/ppl.12134 24286229

[B4] Bishopp A. Benková E. Helariutta Y. (2011a). Sending mixed messages: auxin-cytokinin crosstalk in roots. *Curr. Opin. Plant Biol.* 14 10–16. 10.1016/j.pbi.2010.08.014 20926335

[B5] Bishopp A. Help H. El-Showk S. Weijers D. Scheres B. Friml J. (2011b). A mutually inhibitory interaction between auxin and cytokinin specifies vascular pattern in roots. *Curr. Biol.* 21 917–926. 10.1016/B978-0-323-60984-5.00062-7 21620702

[B6] Bossinger G. Spokevicius A. V. (2018). Sector analysis reveals patterns of cambium differentiation in poplar stems. *J. Exp. Bot.* 69 4339–4348. 10.1093/jxb/ery230 29931329 PMC6093462

[B7] Bouché F. (2018). *2018_Arabidopsis_flowering_plant.* figshare. Figure. 10.6084/m9.figshare.7159937.v1

[B8] Brackmann K. Qi J. Gebert M. Jouannet V. Schlamp T. Grünwald K. (2018). Spatial specificity of auxin responses coordinates wood formation. *Nat. Commun.* 9:875. 10.1038/s41467-018-03256-2 29491423 PMC5830446

[B9] Cammarata J. Roeder A. H. Scanlon M. J. (2019). Cytokinin and CLE signaling are highly intertwined developmental regulators across tissues and species. *Curr. Opin. Plant Biol.* 51 96–104. 10.1016/j.pbi.2019.05.006 31280129

[B10] Chaffey N. Cholewa E. Regan S. Sundberg B. (2002). Secondary xylem development in *Arabidopsis*: a model for wood formation. *Physiol. Plant.* 114 594–600. 10.1034/j.1399-3054.2002.1140413.x 11975734

[B11] De Rybel B. Adibi M. Breda A. S. Wendrich J. R. Smit M. E. Novák O. (2014). Integration of growth and patterning during vascular tissue formation in *Arabidopsis*. *Science* 345:1255215. 10.1126/science.1255215 25104393

[B12] Dolzblasz A. Nardmann J. Clerici E. Causier B. van der Graaff E. Chen J. (2016). Stem cell regulation by *Arabidopsis* WOX genes. *Mol. Plant* 9 1028–1039. 10.1016/j.molp.2016.04.007 27109605

[B13] Eldar A. Elowitz M. B. (2010). Functional roles for noise in genetic circuits. *Nature* 467 167–173. 10.1038/nature09326 20829787 PMC4100692

[B14] Etchells J. P. Mishra L. S. Kumar M. Campbell L. Turner S. R. (2015). Wood formation in trees is increased by manipulating PXY-regulated cell division. *Curr. Biol.* 25 1050–1055. 10.1016/j.cub.2015.02.023 25866390 PMC4406943

[B15] Etchells J. P. Provost C. M. Mishr L. Turner S. R. (2013). WOX4 and WOX14 act downstream of the PXY receptor kinase to regulate plant vascular proliferation independently of any role in vascular organisation. *Development* 140 2224–2234. 10.1242/dev.091314 23578929 PMC3912870

[B16] Etchells J. P. Turner S. R. (2010). The PXY-CLE41 receptor ligand pair defines a multifunctional pathway that controls the rate and orientation of vascular cell division. *Development* 137 767–774. 10.1242/dev.044941 20147378

[B17] Evert R. F. (2006). “Ch. 12 – Vascular cambium ” in *Esau’s Plant Anatomy*, eds Evert R. F. Eichhorn S. E. (Hoboken, NJ: John Wiley & Sons, Ltd). 10.1002/0470047380.ch12

[B18] Fischer U. Kucukoglu M. Helariutta Y. Bhalerao R. P. (2019). The dynamics of cambial stem cell activity. *Annu. Rev. Plant Biol.* 70 293–319. 10.1146/annurev-arplant-050718-100402 30822110

[B19] Fisher K. Turner S. (2007). PXY, a receptor-like kinase essential for maintaining polarity during plant vascular-tissue development. *Curr. Biol.* 17 1061–1066. 10.1016/j.cub.2007.05.049 17570668

[B20] Fukuda H. Ohashi-Ito K. (2019). “Chapter 6 – Vascular tissue development in plants,” in *Plant Development and Evolution*, Vol. 131 ed. Grossniklaus U. (Cambridge, MA: Academic Press), 141–160. 10.1016/bs.ctdb.2018.10.005 30612615

[B21] Greb T. Lohmann J. U. (2016). Plant stem cells. *Curr. Biol.* 26 R816–R821. 10.1016/j.cub.2016.07.070 27623267

[B22] Haecker A. Groß-Hardt R. Geiges B. Sarkar A. Breuninger H. Herrmann M. (2004). Expression dynamics of WOX genes mark cell fate decisions during early embryonic patterning in *Arabidopsis thaliana*. *Development* 131 657–668. 10.1242/dev.00963 14711878

[B23] Ham L. Jackson M. Stumpf M. P. H. (2021). Pathway dynamics can delineate the sources of transcriptional noise in gene expression. *ELife* 10:e69324. 10.7554/eLife.69324 34636320 PMC8608387

[B24] Hirakawa Y. Kondo Y. Fukuda H. (2010). TDIF peptide signaling regulates vascular stem cell proliferation *via* the WOX4 homeobox gene in *Arabidopsis*. *Plant Cell* 22 2618–2629. 10.1105/tpc.110.076083 20729381 PMC2947162

[B25] Hirakawa Y. Shinohara H. Kondo Y. Inoue A. Nakanomyo I. Ogawa M. (2008). Non-cell-autonomous control of vascular stem cell fate by a CLE peptide/receptor system. *Proc. Natl. Acad. Sci. U.S.A.* 105 15208–15213. 10.1073/pnas.0808444105 18812507 PMC2567516

[B26] Huang B. Lu M. Galbraith M. Levine H. Onuchic J. N. Jia D. (2020). Decoding the mechanisms underlying cell-fate decision-making during stem cell differentiation by random circuit perturbation. *J. R. Soc. Interface* 17:20200500. 10.1098/rsif.2020.0500 32781932 PMC7482558

[B27] Immanen J. Nieminen K. Smolander O. P. Kojima M. Alonso Serra J. Koskinen P. (2016). Cytokinin and auxin display distinct but interconnected distribution and signaling profiles to stimulate cambial activity. *Curr. Biol.* 26 1990–1997. 10.1016/j.cub.2016.05.053 27426519

[B28] Ito Y. Fukuda H. (2006). Dodeca-CLE peptides as suppressors. *Science* 313 842–845. 10.1126/science.1128436 16902140

[B29] Larson P. R. (1994). *The Vascular Cambium: Development and Structure.* Berlin: Springer-Verlag.

[B30] Li M. Izpisua Belmonte J. C. (2018). Deconstructing the pluripotency gene regulatory network. *Nat. Cell Biol.* 20 382–392. 10.1038/s41556-018-0067-6 29593328 PMC6620196

[B31] Liebsch D. Sunaryo W. Holmlund M. Norberg M. Zhang J. Hall H. C. (2014). Class I KNOX transcription factors promote differentiation of cambial derivatives into xylem fibers in the *Arabidopsis* hypocotyls. *Development* 141 4311–4319. 10.1242/dev.111369 25371365

[B32] Mähönen A. P. Bishopp A. Higuchi M. Nieminen K. M. Kinoshita K. Törmäkangas K. (2006). Cytokinin signaling and its inhibitor AHP6 regulate cell fate during vascular development. *Science* 311 94–98. 10.1126/science.1118875 16400151

[B33] Masuyama N. Mori H. Yachie N. (2019). DNA barcodes evolve for high-resolution cell lineage tracing. *Curr. Opin. Chem. Biol.* 52 63–71. 10.1016/j.cbpa.2019.05.014 31212208

[B34] Miyashima S. Roszak P. Sevilem I. Toyokura K. Blob B. Heo J. (2019). Mobile PEAR transcription factors integrate positional cues to prime cambial growth. *Nature* 565 490–494. 10.1038/s41586-018-0839-y 30626969 PMC7617008

[B35] Morita J. Kato K. Nakane T. Kondo Y. Fukuda H. Nishimasu H. (2016). Crystal structure of the plant receptor-like kinase TDR in complex with the TDIF peptide. *Nat. Commun.* 7:12383. 10.1038/ncomms12383 27498761 PMC4979064

[B36] Nichols J. Zevnik B. Anastassiadis K. Niwa H. Klewe-Nebenius D. Chambers I. (1998). Formation of pluripotent stem cells in the mammalian embryo depends on the POU transcription factor Oct4. *Cell* 95 379–391. 10.1016/S0092-8674(00)81769-9 9814708

[B37] Okamoto K. Germond A. Fujita H. Furusawa C. Okada Y. Watanabe T. M. (2018). Single cell analysis reveals a biophysical aspect of collective cell-state transition in embryonic stem cell differentiation. *Sci. Rep.* 8:11965. 10.1038/s41598-018-30461-2 30097661 PMC6086879

[B38] Peterson V. M. Zhang K. X. Kumar N. Wong J. Li L. Wilson D. C. (2017). Multiplexed quantification of proteins and transcripts in single cells. *Nat. Biotechnol.* 35 936–939. 10.1038/nbt.3973 28854175

[B39] Randall R. S. Miyashima S. Blomster T. Zhang J. Elo A. Karlberg A. (2015). AINTEGUMENTA and the D-type cyclin CYCD3;1 regulate root secondary growth and respond to cytokinins. *Biol. Open* 4 1229–1236. 10.1242/bio.013128 26340943 PMC4610221

[B40] Roszak P. Heo J.-O. Blob B. Toyokura K. Sugiyama Y. de Luis Balaguer M. A. (2021). Cell-by-cell dissection of phloem development links a maturation gradient to cell specialization. *Science* 374:eaba5531. 10.1126/science.aba5531 34941412 PMC8730638

[B41] Růzǐčka K. Šimášková M. Duclercq J. Petrášek J. Zažímalová E. Simon S. (2009). Cytokinin regulates root meristem activity via modulation of the polar auxin transport. *Proc. Natl. Acad. Sci. U.S.A.* 106 4284–4289. 10.1073/pnas.0900060106 19246387 PMC2657394

[B42] Sachs T. (1991). Cell polarity and tissue patterning in plants. *Devlopment* 91 83–93.

[B43] Salvi E. Rutten J. P. Di Mambro R. Polverari L. Licursi V. Negri R. (2020). A self-organized PLT/Auxin/ARR-B network controls the dynamics of root zonation development in *Arabidopsis thaliana*. *Dev. Cell* 53 431–443.e23. 10.1016/j.devcel.2020.04.004 32386600

[B44] Sanio K. G. (1873). Anatomie der gemeinen Kiefer (*Pinus sylvestris* L.). *Jahrb. Wiss. Bot.* 9 50–126.

[B45] Shi D. Jouannet V. Agustí J. Kaul V. Levitsky V. Sanchez P. (2021). Tissue-specific transcriptome profiling of the *Arabidopsis* inflorescence stem reveals local cellular signatures. *Plant Cell* 33 200–223. 10.1093/PLCELL/KOAA019 33582756 PMC8136906

[B46] Shi D. Lebovka I. Loṕez-Salmeroń V. Sanchez P. Greb T. (2019). Bifacial cambium stem cells generate xylem and phloem during radial plant growth. *Development* 146:dev171355. 10.1242/dev.171355 30626594 PMC6340147

[B47] Šimášková M. O’Brien J. A. Khan M. Van Noorden G. Ötvös K. Vieten A. (2015). Cytokinin response factors regulate PIN-FORMED auxin transporters. *Nat. Commun.* 6:8717. 10.1038/ncomms9717 26541513

[B48] Smet W. Sevilem I. de Luis Balaguer M. A. Wybouw B. Mor E. Miyashima S. (2019). DOF2.1 controls cytokinin-dependent vascular cell proliferation downstream of TMO5/LHW. *Curr. Biol.* 29 520–529.e6. 10.1016/j.cub.2018.12.041 30686737 PMC6370950

[B49] Smetana O. Mäkilä R. Lyu M. Amiryousefi A. Sánchez Rodríguez F. Wu M. F. (2019). High levels of auxin signalling define the stem-cell organizer of the vascular cambium. *Nature* 565 485–489. 10.1038/s41586-018-0837-0 30626967

[B50] Smit M. E. McGregor S. R. Sun H. Gough C. Bågman A. M. Soyars C. L. (2020). A PXY-mediated transcriptional network integrates signaling mechanisms to control vascular development in *Arabidopsis*. *Plant Cell* 32 319–335. 10.1105/tpc.19.00562 31806676 PMC7008486

[B51] Su Y. H. Liu Y. B. Zhang X. S. (2011). Auxin-cytokinin interaction regulates meristem development. *Mol. Plant* 4 616–625. 10.1093/mp/ssr007 21357646 PMC3146736

[B52] Suer S. Agusti J. Sanchez P. Schwarz M. Greb T. (2011). WOX4 imparts auxin responsiveness to cambium cells in *Arabidopsis*. *Plant Cell* 23 3247–3259. 10.1105/tpc.111.087874 21926336 PMC3203433

[B53] Turley E. K. Etchells J. P. (2021). Laying it on thick: a study in secondary growth. *J. Exp. Bot.* 73 665–679. 10.1093/jxb/erab455 34655214 PMC8793872

[B54] Uggla C. Moritz T. Sandberg G. Sundberg B. (1996). Auxin as a positional signal in pattern formation in plants. *Proc. Natl. Acad. Sci. U.S.A.* 93 9282–9286. 10.1073/pnas.93.17.9282 11607701 PMC38633

[B55] Wallner E. S. López-Salmerón V. Belevich I. Poschet G. Jung I. Grünwald K. (2017). Strigolactone- and karrikin-independent SMXL proteins are central regulators of phloem formation. *Curr. Biol.* 27 1241–1247. 10.1016/j.cub.2017.03.014 28392107 PMC5405109

[B56] Wallner E. S. Tonn N. Shi D. Jouannet V. Greb T. (2020). SUPPRESSOR OF MAX2 1-LIKE 5 promotes secondary phloem formation during radial stem growth. *Plant J.* 102 903–915. 10.1111/tpj.14670 31910293

[B57] Yang B. J. Minne M. Brunoni F. Plačková L. Petřík I. Sun Y. (2021). Non-cell autonomous and spatiotemporal signalling from a tissue organizer orchestrates root vascular development. *Nat. Plants* 7 1485–1494. 10.1038/s41477-021-01017-6 34782768 PMC7612341

[B58] Ye L. Wang X. Lyu M. Siligato R. Eswaran G. Vainio L. (2021). Cytokinins initiate secondary growth in the *Arabidopsis* root through a set of LBD genes. *Curr. Biol.* 31 3365–3373.e7. 10.1016/j.cub.2021.05.036 34129827 PMC8360765

[B59] Zhang H. Lin X. Han Z. Qu L. J. Chai J. (2016). Crystal structure of PXY-TDIF complex reveals a conserved recognition mechanism among CLE peptide-receptor pairs. *Cell Res.* 26 543–555. 10.1038/cr.2016.45 27055373 PMC4856767

[B60] Zhang J. Eswaran G. Alonso-Serra J. Kucukoglu M. Xiang J. Yang W. (2019). Transcriptional regulatory framework for vascular cambium development in *Arabidopsis* roots. *Nat. Plants* 5 1033–1042. 10.1038/s41477-019-0522-9 31595065 PMC6795544

[B61] Zhang M. Zou Y. Xu X. Zhang X. Gao M. Song J. (2020). Highly parallel and efficient single cell mRNA sequencing with paired picoliter chambers. *Nat. Commun.* 11:2118. 10.1038/s41467-020-15765-0 32355211 PMC7193604

[B62] Zhou Y. Liu X. Engstrom E. M. Nimchuk Z. L. Pruneda-Paz J. L. Tarr P. T. (2015). Control of plant stem cell function by conserved interacting transcriptional regulators. *Nature* 517 377–380. 10.1038/nature13853 25363783 PMC4297503

